# Do Daily and Seasonal Changes in Non‐Structural Carbohydrates in Grapevine Leaves Contribute to Osmotic Adjustment and Regulation of Photosynthesis?

**DOI:** 10.1111/ppl.70683

**Published:** 2025-12-29

**Authors:** Aviad Perry, Or Sperling, Alon Ben‐Gal, N. Michele Holbrook, Shimon Rachmilevitch, Uri Hochberg

**Affiliations:** ^1^ Kreitman School for Graduate Studies Ben‐Gurion University of the Negev Be'er Sheva Israel; ^2^ Plant Sciences Agricultural Research Organization (ARO) ‐ Volcani, Gilat Research Center Gilat Israel; ^3^ Soil, Water, and Environmental Sciences Agricultural Research Organization (ARO) ‐ Volcani, Gilat Research Center Gilat Israel; ^4^ Organismic and Evolutionary Biology Harvard University Cambridge Massachusetts USA; ^5^ French Associates Institute for Agriculture and Biotechnology of Drylands, the Jacob Blaustein Institutes for Desert Research Ben Gurion University of the Negev Midreshet Ben Gurion Israel; ^6^ Soil, Water, and Environmental Sciences Agricultural Research Organization (ARO) ‐ Volcani, Neve Ya'ar Research Center Ramat Yishai Israel

**Keywords:** drought, grapevines, NSC, osmotic adjustment, photosynthesis

## Abstract

Leaves maintain a pool of non‐structural carbohydrates (NSC) whose size can vary over hourly and longer timescales. We tested two long‐standing hypotheses regarding potential physiological roles of changes in foliar NSC levels. The first is that soluble NSC plays a critical role in osmotic adjustment, with their increase enabling stomatal opening despite daily and seasonal reductions in leaf water potential (*Ψ*_leaf_). The second is that increases in NSC are a sign of excess assimilation relative to sink demand and serve as a signal to downregulate gas exchange. To explore these questions, we monitored the diurnal and seasonal dynamics of gas exchange, *Ψ*_leaf_, osmotic potential, and NSC of irrigated and dehydrated grapevines ( *Vitis vinifera* ) through two consecutive growing seasons. We found that the daily accumulation of soluble sugars constitutes approximately 50% of the daily osmotic adjustment (0.2 MPa), enabling the vines to maintain turgor under low *Ψ*_leaf_. At the same time, the importance of NSC as osmolytes decreased as the season progressed, and they did not contribute to osmotic adjustments when water was withheld. Additionally, there was no negative correlation between NSC and gas exchange, implying that bulk NSC concentration is not the signal for photosynthetic feedback inhibition.

## 1 Introduction

Non‐structural carbohydrates (NSC) are the primary form of stored energy in plants. Their synthesis largely takes place in leaves, with most being promptly transported to ‘sink organs’ through the phloem. Only a small fraction of the NSC produced each day remains in the leaves (Davie et al. 2000), where they serve as a local energy source. Because the leaf NSC content is small compared to the daily NSC flux, minor mismatches between photosynthesis and phloem export lead to significant changes in the leaf NSC content within only a few hours (Acevedo et al. 1979; Gersony et al. 2020). These fast dynamics have led to hypotheses that, in addition to their energetic role, foliar NSC could serve for osmotic adjustment (Munns and Weir 1981; Long and Adams 2023) and possibly act as signaling molecules for gas exchange regulation (Azcón‐Bieto 1983; Zait et al. 2025). These two long‐standing hypotheses have mostly been tested under short‐term experiments and using a limited set of species, making it difficult to generalize their validity among taxa or environmental conditions.

### 1.1 NSC as Important Osmolytes

Soluble carbohydrates (SC) comprise a large proportion of leaf NSC and thus can be major contributors to leaf osmotic potential. In most species, SC makes up most of the organic solutes (Arndt et al. 2000; Lannucci et al. 2002), and in grapevines, they were reported to constitute 50%–70% of the total solutes (Rodrigues et al. 1993; Patakas et al. 2002). This high proportion, and the multiple studies that found an increase in leaf SC content under water deficit conditions (Cabuslay et al. 2002; O'Brien et al. 2015; Vuerich et al. 2021), has led to the hypothesis that sugars are critical for osmotic adjustment (Turner and Begg 1981). In support, several studies found that the increase in SC concentration following water deficit composed most of the osmotic adjustment (Munns and Weir 1981; Kameli and Lösel 1995; Clifford et al. 1998). The rationale for this hypothesis is that since plant growth is more sensitive to water deficits than photosynthesis, drought should lead to sugar accumulation (Munns 1988; Muller et al. 2011).

However, the hypothesis of SC contribution to osmotic adjustment does not hold under all conditions. Several studies found that sugars constituted less than 30% of the total osmotic adjustment (Jones et al. 1980; Itoh and Kumura 1987; Sánchez et al. 1998; da Silva and Arrabaça 2004), while others reported that sugars did not contribute to osmotic adjustment at all (Lannucci et al. 2002; Patakas et al. 2002; Ottow et al. 2005). Intrinsic differences between species could partly explain this variability. However, the fact that SC contribution to osmotic adjustment was different even in the same species under different experimental settings (Munns and Weir 1981; Kameli and Lösel 1995; Hu and Schmidhalter 1998; Bajji et al. 2001) suggests that it is also affected by environmental conditions or plant phenology.

SC also appears to play a critical role in the daily dynamics of leaf water status. The accumulation of SC during photosynthetic hours could represent a diurnal osmotic adjustment that enables plants to maintain turgor at midday when their water potential is minimal (Acevedo et al. 1979; Gersony et al. 2020; Traversari et al. 2020). Surprisingly, to our knowledge, this contribution has not been tested under water deficit conditions when the challenge of turgor maintenance is greatest. Since water deficit typically induces significantly lower photosynthetic rates, it is unclear if SC synthesis is sufficient to buffer the sizeable daily drop in water potential.

### 1.2 NSC as Signals Regulating Gas Exchange

Over 150 years ago, Boussingault (1868) hypothesized that the accumulation of assimilates in a leaf might be responsible for downregulating its photosynthesis (Goldschmidt and Huber 1992). This hypothesis arises from the perception that the leaf carbon content (as NSC) reflects the plant's sink‐source balance and could signal photosynthetic adjustment to better align the production of carbohydrates with their demand. Dozens of studies supported Boussingault's hypothesis (e.g., Azcón‐Bieto 1983; Layne and Flore 1995; McCormick et al. 2008; Piccolo et al. 2020), with some even suggesting signaling pathways (Koch et al. 1996; Kelly et al. 2013, 2014). Accordingly, feedback inhibition by photosynthetic end‐products (NSC) is incorporated into many plant water‐carbon models (Gent and Seginer 2012; Hölttä et al. 2017; Dewar et al. 2022).

However, it is important to mention that nearly all of the attestations for the involvement of NSC in gas exchange regulation were acquired by crude source‐sink manipulation, such as girdling (Piccolo et al. 2020), fruit removal (Goldschmidt and Huber 1992), sugar feeding (Kottapalli et al. 2018), exposure to high CO_2_ (Sawada et al. 2001), severe chilling (Azcón‐Bieto 1983), or defoliation (Layne and Flore 1995). As far as we can tell, there are no reports concerning the association between photosynthesis and leaf NSC content under normal conditions. The main knowledge gap, in this respect, is due to the limited leaf NSC measurements that can be compared against measurements of photosynthesis.

Hence, we set out to test the hypotheses that NSC contributes to osmotic adjustment and assess their inhibitory effects on photosynthesis. Our goal was to create a substantial dataset of grapevine leaf NSC concentrations throughout the day and growing season and compare it to their osmotic content and photosynthetic rates. We postulated that if we monitored vines long enough, we would find considerable variability in foliar NSC, allowing us to examine their relationship with leaf osmolarity and photosynthesis. We also subjected the vines to episodic droughts during the growing season to further increase the variability in leaf NSC contents and test the hypotheses under stress conditions.

## 2 Materials and Methods

We monitored vine physiological activity and sugar content through two consecutive growing seasons (2020 and 2021) to associate daily and seasonal carbon balance of leaves with photosynthetic regulation and osmotic adjustments.

Experimental setup:

The experiment was conducted at the Gilat Agricultural Research Center located in the northern Negev Desert in Israel, which is characterized by a semi‐arid Mediterranean climate with an average precipitation of about 250 mm y^−1^. Rainfall occurs almost exclusively in winter from November to March, while the summer is dry and hot with no precipitation. Typically, in summer, the temperatures vary between 35°C during the day and 20°C at night. Specific environmental conditions of the experimental years can be found in Figures S2–S4.

Eight vines (“Early Sweet” grafted on “140 Ruggeri”) were planted in 10 L pots in March 2018. In March 2020, the vines were transplanted into 2 m^3^ (1.4 m diameter and 1.3 m height) containers filled with sandy loam soil (88%–95% sand and 4%–10% clay). The containers had a rockwool drainage extension (Ben‐Gal and Shani 2002) that excluded saturation at the lower soil boundary and enabled drainage collections. Y‐shaped trellises were installed in the containers to mechanically support canopy growth within an area of 1.4 by 2.7 m (Figure S1). During the experiment, the vines were three (in 2020) or four (in 2021) years old. The pots were placed outdoors with no shading or cover. The vines were pruned during dormancy (March 2020 and March 2021) and trimmed 4–5 times during summer to maintain their canopy within the trellises. The vines were harvested on June 28th in 2020 and June 24th in 2021. Vines were continuously fertigated with mineral fertilizers (40 mg N l^−1^, 10 mg P l^−1^, 60 mg K l^−1^, 35 mg Ca l^−1^, 15 mg Mg l^−1^, 0.016 mg Mo l^−1^, 0.025 mg Cu l^−1^, 0.15 mg Zn l^−1^, 0.3 mg Mn l^−1^, and 0.6 mg Fe l^−1^) which corresponded to commercial vineyard management.

Vines were drip irrigated (16 mm pipe with nine 1.2 L h^−1^ compensated drippers) daily to 130% of their water uptake based on daily mass balance between irrigation and daily drainage collections. Once a month, we dehydrated four of the vines (always the same four) by withholding the water completely until their midday stomatal conductance (g_s_) was lower than 30 mmol m^−2^ s^−1^. In 2020, the dehydration process lasted 7 days in June and 8 days in July or August. In 2021, the dehydration process lasted 6 days in June and 7 days in July and August. In total, the four vines went through six dehydration periods.

### 2.1 Physiological Measurements

Starting 4–5 weeks after bud break (Mid May 2020 or last week of April 2021), we measured the daily dynamics of multiple parameters, including gas exchange, leaf water potential, leaf mass per area, the leaf osmotic potential, and sugar concentration. This set of parameters was assessed every ~14 days, resulting in eight measurement days in 2020 and seven measurement days in 2021 (Table S1). Measurements were conducted only on cloudless days. On each measurement day, this set of parameters was measured from predawn until after sunset (seven sampling points: 04:30, 06:30, 09:00, 12:00, 15:00, 18:00, and 20:30). During the six droughts, midday gas exchange (irrigated and stressed vines) was recorded once a day (at midday) to monitor the dehydration process. The diurnal physiological pattern was measured on the last day of the dehydration.

### 2.2 Gas Exchange

Gas exchange was measured for two sun‐exposed (during the day), youngest, fully expanded leaves on two different shoots in each vine by a CIRAS‐3 portable photosynthesis system (PP Systems). CO_2_ was fixed at 400 ppm and relative humidity at 70% of ambient conditions. The light was provided by LEDs (red 90%–blue 10%), and their intensity was adjusted to the solar irradiance at the beginning of each measurement. The flow was set to 350 cc min^−1^. Values were logged only after stability was reached (approximately 2 min after clamping the leaf).

### 2.3 Leaf Water Potential

A single mature undamaged leaf from the upper part of the stem was cut from each plant at the petiole into a foil bag for *Ψ*_leaf_ measurement. At the lab, the leaf water potential was measured within 15 min from excision (Hochberg 2020) using a pressure chamber (model 600D, PMS instruments Co.). The leaf was placed into the pressure chamber with the petiole cut protruding out. The pressure was increased at 0.01 MPa s^−1^ and the result was determined at the first site of sap rising into the cut surface.

### 2.4 Leaf Dry Mass Per Area

To examine the total accumulation of matter along the day, we followed the daily leaf mass per area (LMA). At each sampling point, six pre‐marked leaves per plant were sampled by cutting one disc per leaf (using a 6 mm paper hole punch) into an Eppendorf tube. The discs were dried (3 days in a 70°C oven) and weighed for dry mass so that LMA could be calculated:

(1) $$\mathrm{LMA}\;\left(\mathrm{mg}\;m^{-2}\right)=\frac{\text{average disc mass}\;\left(\mathrm{mg}\right)}{\text{disc area}\;\left(m^{2}\right)}$$

### 2.5 Metabolic Measurements

Leaves used for *Ψ*_leaf_ measurements were also sampled for NSC and solute concentration (*π*) analysis (tested only in 2021). Each leaf was cut in half (excluding the mid vein), and each half was placed into 1.5 mL (for *π*) or 2 mL (for NSC) Eppendorf tubes for immediate freezing in liquid nitrogen to stop all metabolism. The NSC samples were then dried for 3 days in the oven (70°C), while the *π* samples remained in a −20°C freezer until analysis.

### 2.6 Total Solute Concentration and Osmotic Potential

Liquid for *π* measurements was collected by punching a hole in the bottom of the 1.5 mL Eppendorf tube using a hot needle, placing it inside a 2 mL Eppendorf tube (for collecting the liquid), and centrifuging it for 3 min at 14000 g. Leaf samples were defrosted for 5 min before centrifugation. The concentration of osmotically active solutes (mmol kg^−1^) was determined by vapor pressure osmometry (VAPRO 5520 and 5600; Wescor Inc).

Solute concentration was converted to osmotic potential (*Ψπ*) using the van't‐Hoff equation:

(2) $$\Psi \pi =\frac{-\mathrm{\pi RT}}{1-A_{f}}$$

where *π* is the solute concentration (mmol kg^−1^), *R* is the universal gas constant (8.314 × 10^−6^ m^3^ MPa K^−1^ mol^−1^), *T* is the temperature (assumed as 298 K), and *A*_*f*_ is the apoplastic fraction assumed to be 10% (Arndt et al. 2015).

### 2.7 Non‐Structural Carbohydrates

NSC concentrations (mg per g dry leaf material) were determined by colorimetry (with anthrone) following Sperling et al. (2017). In short, 25 mg of powder from oven‐dried leaves was weighed into a 1.5 mL Eppendorf tube, diluted in 1 mL of double distilled water (DDW), and incubated for 15 min at 72°C. After centrifuging, 50 μL of the supernatant was taken for determination of the glucose‐equivalent SC concentration in 96% sulfuric acid and anthrone at 620 nm absorption. The remaining pellet was washed twice in DDW and mixed with 500 μL of 0.2 M Na acetate buffer (pH 5.5), 100 μL of amyloglucosidase (70 units ml^−1^), and 100 μL of *α*‐amylase (7 units ml^−1^). The mixture was incubated for 4 h at 37°C. After another centrifuging at 14000 g, 50 μL of the supernatant was taken for the glucose‐equivalent starch determination.

Conversion of the NSC weight fraction (g g^−1^) into concentration per leaf area (g m^−2^) was performed using the average LMA (Equation 1) of all the measured leaf discs (an average of 40 g m^−2^; *n* = 392).

The osmotic concentration of SC (*π*SC, mmol per kg of water) was estimated according to:

(3) $$\pi \mathrm{SC}\;\left(\text{mmol}\;{\mathrm{kg}}^{-1}\right)=\frac{\mathrm{SC}\;\left(\mathrm{mg}\;g^{-1}\right)\times \frac{\mathrm{DM}\;}{\mathrm{WM}}\;}{M-\mathrm{SC}\;\left(g\;{\mathrm{mol}}^{-1}\right)}$$

The DM/WM ratio is the relative proportion between the dry leaf mass and leaf water mass (1/3 as measured by oven drying leaves). M‐SC is the SC molar mass based on Rodrigues et al. (1993), who reported that grapevine leaves SC composition is 38% glucose and fructose (180.16 g mol^−1^) and 62% sucrose (342.3 g mol^−1^).

### 2.8 Data Analysis

Data were processed using *base R* (R Core Team 2021) and the data. Table package (Dowle and Srinivason 2021) in the RStudio environment (Allaire 2012). Statistics were completed by the *agricolae* package (de Mendiburu 2021) and illustrated with the *ggplot2* package (Wickham 2016).

To assess the effects of dehydration, treatment, and time of day on leaf physiological traits, we fitted linear mixed effects models for each response variable (A_N_, g_s_, SC, Starch, *Ψ*_leaf_, and *π*). For each trait, the model took the form:

$$Y∼\text{time}\times \text{treatment}\times \text{season}+\left(1|\text{date}\right)$$

where *time* (measurement hour) *treatment* (Irrigated vs. dehydrated vines) and season (2020 vs. 2021) were treated as fixed factors, and *date* (measurement day) was included as a random intercept to account for repeated measurements within the same day.

All models were fitted using the *lme4* package (Bates et al. 2015), with type III tests of fixed effects obtained using the *lmerTest* package (Kuznetsova et al. 2017).

To understand the effect of dehydration treatment on physiological factors (*A*_N_, g_s_, *Ψ*_leaf_, SC, starch, and *π*) at a given hour, we used two‐tailed Student t‐tests, comparing all the repetitions from the irrigated and dehydrated treatments. We analyzed differences among measurement days and hours using a one‐way ANOVA. When significant effects were detected, pairwise comparisons were performed using Tukey's Honestly Significant Difference (HSD) test to control for multiple testing. For the comparison of diurnal changes in *A*_N_, g_s_, *Ψ*_leaf_, SC, starch, and *π*, we used the Tukey HSD test, comparing all the repetitions along the season in a given hour to other hours of the day in the same treatment. For the comparison of SC contribution to total osmotic concentration, we used the Tukey HSD test, comparing every measurement day with all the other measurement days in 2021.

To test the relations between NSC and A_N_ or gs, we tested the significance of a linear regression of either all the hours pulled together or separated into the different hours of the day.

## 3 Results

Our mixed model suggested that the irrigation treatment and the time of day had a significant effect on all the physiological factors, apart from SC and *π* that were significantly affected by the time of day, but not by the treatment (Table 1). The 2 years of the experiment did not have much of an effect on the results. There was also a clear interaction between the time of day and irrigation treatment for most of the physiological parameters. More details on the statistical model can be found in Table S2.

**TABLE 1:** Summary of type III ANOVA *p*‐values for linear mixed‐effects models testing the effects of measurement time, treatment, and season on physiological traits: Assimilation rate (A_N_), stomatal conductance (g_s_), leaf water potential (*Ψ*_leaf_), soluble carbohydrate (SC), starch, and osmotic concentration (*π*).

Variable	Time	Treatment	Season	Time: treatment	Time: season	Treatment: season	Time: treatment:season
A_N_	< 0.001***	< 0.001***	0.054	< 0.001***	< 0.001***	< 0.001***	0.001**
g_s_	< 0.001***	< 0.001***	0.998	< 0.001***	0.267	0.020 *	0.176
*Ψ*_leaf_	< 0.001***	< 0.001***	0.020 *	< 0.001***	< 0.001***	< 0.001***	< 0.001***
SC	< 0.001***	0.881	0.285	0.299	0.485	0.295	0.91
Starch	< 0.001***	< 0.001***	0.282	< 0.001***	0.022*	< 0.001***	0.559
*π*	0.002**	0.14		0.847			

*Note:* For osmotic concentration, which was measured only in the 2021 season, the model included time and treatment as fixed factors and excluded season. Reported are *p*‐values for each main effect and interaction. Significance levels: *p ≤ 0.05, **p ≤ 0.01, ***p ≤ 0.001. More details on the model results appear in the Supporting Information (Table S2).

### 3.1 Water Potential and Gas Exchange

**FIGURE 1:**
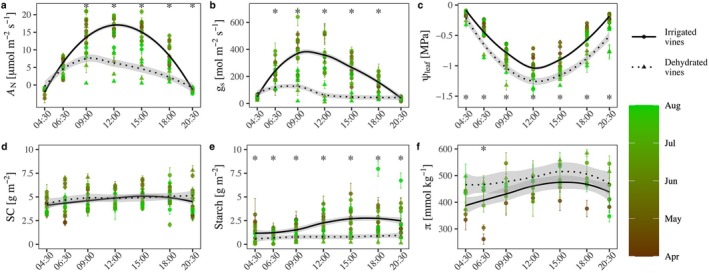
Daily patterns along the growth season of carbon assimilation (a; A_N_), stomatal conductance (b; g_s_), leaf water potential (c; *ψ*_leaf_), leaf soluble carbohydrate content (d; SC), leaf starch content (e), and osmotic concentration (f; *π*). The color scale represents the measurement date, changing from black (April) to light green (August). We present the measurements of the dehydrated vines only during the three droughts per year. Accordingly, the data is composed of 15 measurement days for the irrigated vines (circle) and six measurement days for dehydrated vines (triangle) in 2020 and 2021. Each point represents the mean ± SE of four plants. The lines represent the best fit of the means of each treatment (solid = irrigated vines, dashed = dehydrated). Statistical analysis of each treatment diurnal pattern appears in Table 2. Asterisks represent significant differences between means of treatments.

Initially, we set out to characterize diurnal changes in gas exchange and *Ψ*_leaf_ throughout the growing season. In April, irrigated vines reached daily peak net carbon assimilation (*A*_N_) rates of 16.5 μmol m^−2^ s^−1^ by noon, while dehydrated vines peaked at 8 μmol m^−2^ s^−1^ at 9:00 (Figure 1a, *p* ≦ 0.05). The diurnal assimilation pattern, and the differences between irrigated and dehydrated vines, persisted throughout the summer. In April and May, irrigated vines reached maximal stomatal conductance (g_s_) of 390 mmol m^−2^ s^−1^ by 09:00, sustained it until noon, and reduced g_s_ gradually during the afternoon (Figure 1b). Later in the season, irrigated vines reduced g_s_ by noon. In contrast, dehydrated vines had a lower maximal g_s_ (seasonal average of 171 ± 78 mmol m^−2^ s^−1^), which was reached at a much earlier time of the day (06:30). The dehydrated vines maintained low g_s_ of 15–70 mmol m^−2^ s^−1^ throughout the afternoon (12:00 to 20:30).

The seasonal average leaf water potential (*Ψ*_leaf_) of the irrigated vines was −0.12 ± 0.02 MPa at 04:30, decreasing until noon to ~−1 MPa, and increasing during the afternoon (Figure 1c). Irrigated vines gradually reduced daily minimal *Ψ*_leaf_ throughout the season, beginning at −0.86 MPa in April and decreasing to −1.27 MPa in August. The dehydrated vines exhibited 0.1–0.3 MPa lower *ψ*_leaf_ than irrigated vines throughout both individual measurement days and across the growing season.

### 3.2 Diurnal and Seasonal Variation in NSC

SC exhibited a more subtle daily pattern compared with gas exchange. On average, the SC of irrigated vines was ~4 g m^−2^ at 04:30, increased to ~5 g m^−2^ by 15:00, and then declined to ~4.7 g m^−2^ at 20:30 (Figure 1d). SC of dehydrated vines also increased from a pre‐dawn value of ~4 g m^−2^ to ~5 g m^−2^ at 15:00 and then remained constant throughout the afternoon, though differences weren't significant (Figure 1d; Table 2). Maximum SC levels of irrigated vines declined seasonally from 6.2 g m^−2^ in April to 4.04 g m^−2^ by August, while dehydrated vines sustained a 5 g m^−2^ peak concentration throughout the growing season.

**TABLE 2:** Statistical comparison of diurnal changes in physiological parameters.

Treatment	Variable	4:30	6:30	9:00	12:00	15:00	18:00	20:30
Irrigated vines	A_N_	c	b	a	a	a	b	c
	g_s_	d	b	a	a	b	c	d
	*Ψ*_leaf_	a	bc	d	e	de	c	ab
	SC	b	b	ab	ab	a	a	ab
	Starch	c	c	bc	ab	a	a	a
	*π*	bc	c	abc	abc	a	ab	abc
Dehydrated vines	A_N_	c	ab	a	ab	ab	bc	c
	g_s_	b	a	a	b	b	b	b
	*Ψ*_leaf_	a	ab	cd	d	d	bc	ab
	SC	a	a	a	a	a	a	a
	Starch	a	a	a	a	a	a	a
	*π*	a	a	a	a	a	a	a

*Note:* Tukey HSD comparison of seasonal averages of diurnal change of carbon assimilation (A_N_), stomatal conductance (g_s_), leaf water potential (*ψ*_*leaf*_), leaf soluble carbohydrate content (SC), leaf starch content, and osmotic concentration (*π*) in irrigated and dehydrated vines. The data is composed of 15 measurement days for the irrigated vines and six measurement days for dehydrated vines in 2020 and 2021. Different letters represent statistically significant differences between hours (*p* ≦ 0.05).

The average starch level of irrigated vines was 1.2 g m^−2^ at 04:30, increasing by 118% to 2.65 g m^−2^ at 15:00 and remaining at this concentration until sunset (Figure 1e). The starch concentration of the dehydrated vines was much lower both in terms of absolute values and daily accumulation. The dehydrated vines' starch concentration was half (0.63 g m^−2^) of the starch levels in the irrigated vines at 04:30. By 18:00, the dehydrated vines' starch levels increased by only 55%, to 0.98 g m^−2^, then decreased slightly to 0.91 g m^−2^ by 20:30.

### 3.3 Soluble Carbohydrates and Osmotic Adjustment

**FIGURE 2:**
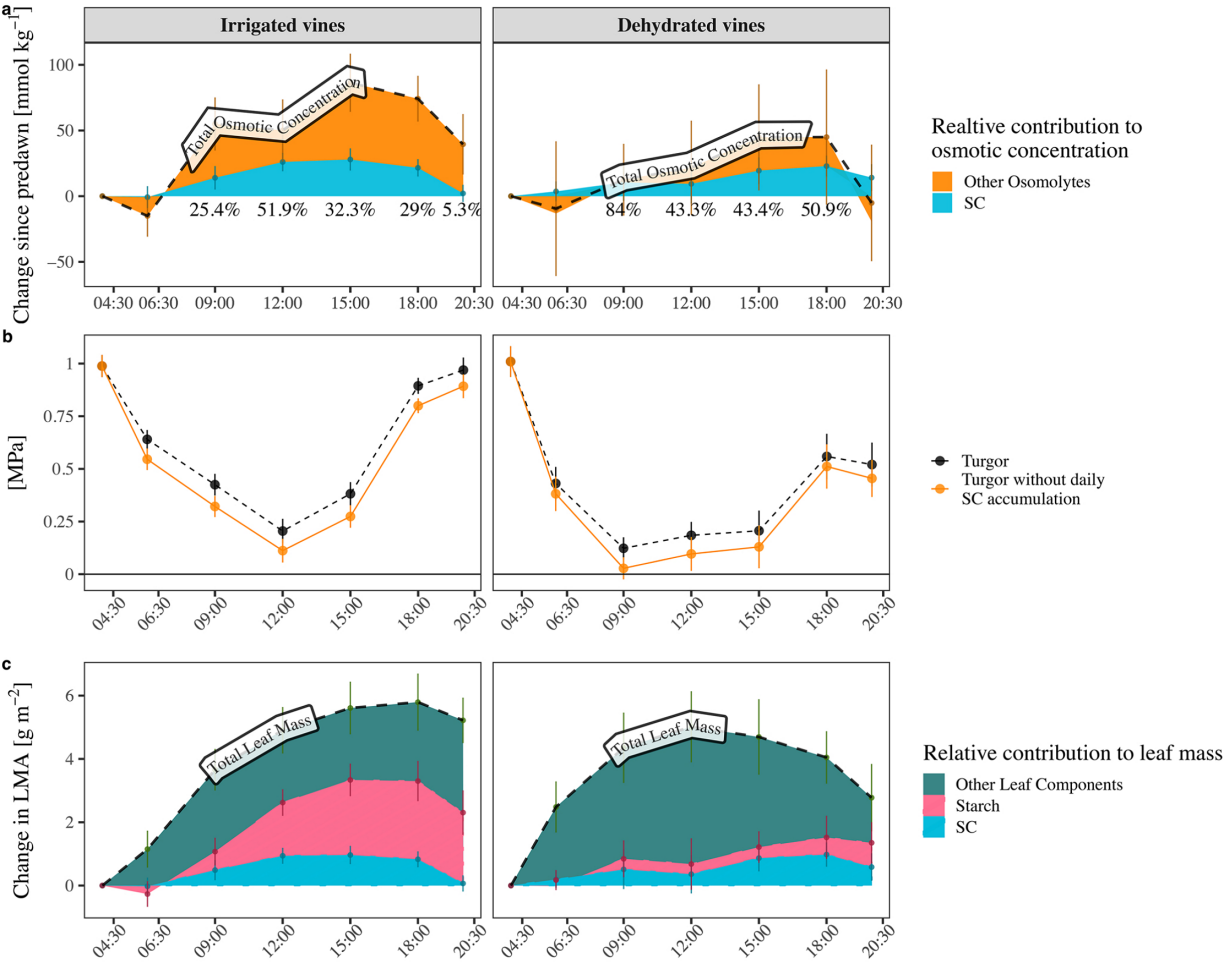
The relative contribution of SC (blue) and other osmolytes (orange) to the change in total osmotic concentration since predawn (dashed line) (a) and the contribution of SC to turgor, presented as turgor with SC (black) and without SC (orange) (b). The relative contribution of starch (pink), SC (blue), and other leaf components (green‐orange stripes) to the daily change in leaf mass per area (dashed line) since predawn (c). Each point represents the mean ± SE of four vines.

Similar to the diurnal pattern of sugar accumulation, the osmotic concentration (*π*) of irrigated vines increased during the morning and peaked at 483 ± 96 mmol kg^−1^ by 15:00. The osmotic concentration of dehydrated vines was higher than that of the irrigated vines by 50–100 mmol kg^−1^ throughout the day, reaching a daily maximum of 460–610 mmol kg^−1^ at 18:00 (Figure 1f). SC played a central role in the daily osmotic adjustment, composing 32.3% of the daily *π* accumulation in the irrigated vines and 50.9% in the dehydrated vines (Figure 2a). The differences between the treatments in SC contribution to *π* were not significant. Accumulated SC (transformed to osmotic potential) contributed up to 0.077 MPa (at 15:00) to the hydrated vines' turgor and up to 0.063 MPa (at 18:00) to the dehydrated vines' turgor (Figure 2b). Without this daily accumulation of SC, the dehydrated vines would have lost their turgor at 9:00. We also compared the NSC accumulation to the daily change in LMA. The daily change in LMA of irrigated vines peaked at 5.79 g m^−2^ by 18:00. In contrast, dehydrated vines' change in LMA peaked at noon with 5.01 g m^−2^ (Figure 2c). To see how much of that daily LMA change was driven by NSC, we compared the expected mass change based on NSC alone with the LMA change. NSC comprised 57% of the maximal leaf mass change in the irrigated plants and a lower proportion (13%) in the dehydrated ones. In the irrigated vines, starch was the main contributor to the daily increase in NSC mass. Irrigated vines accumulated 2.47 g m^−2^ of starch from predawn to the daily peak, while dehydrated vines accumulated only 0.32 g m^−2^.

**FIGURE 3:**
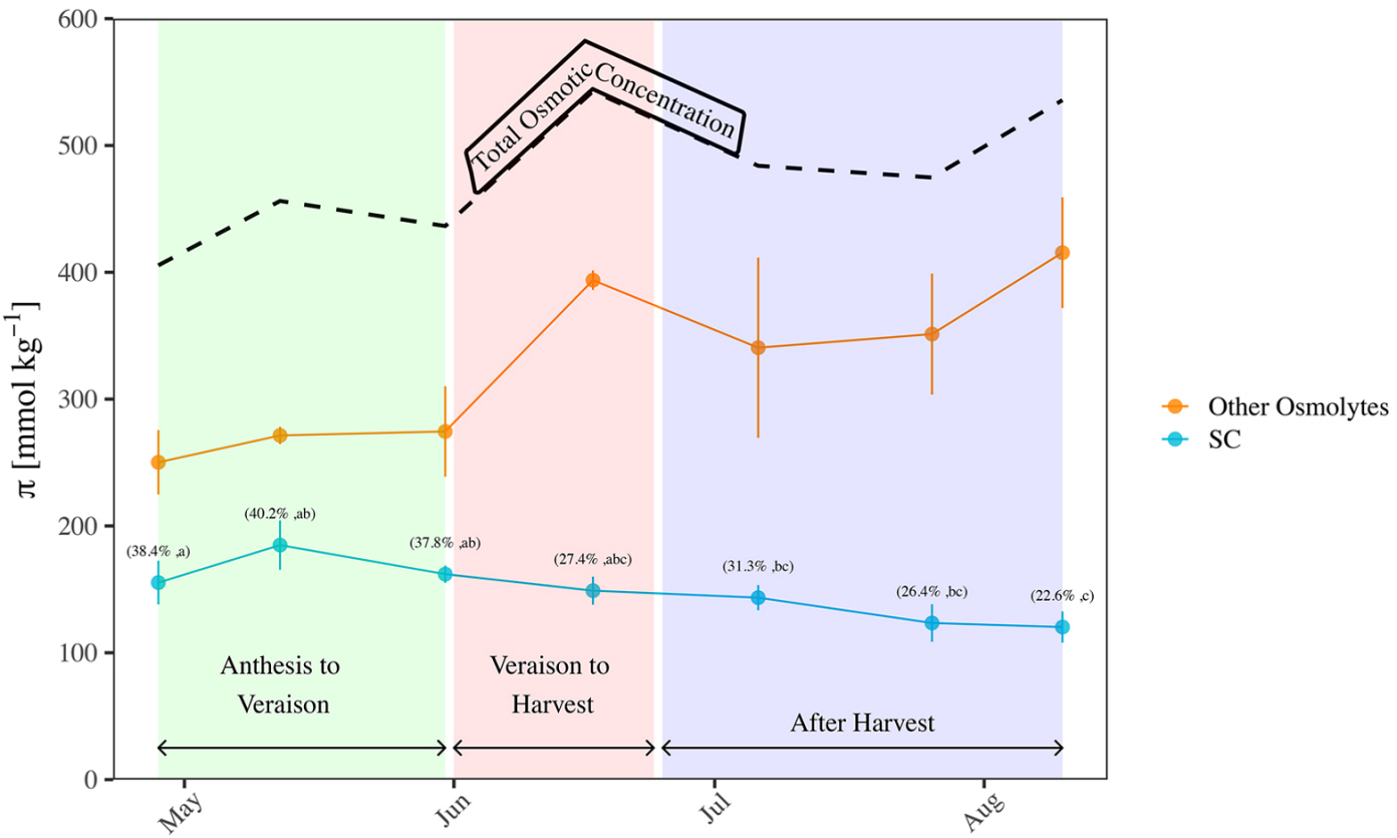
Seasonal pattern of osmotic concentration of soluble carbohydrates (blue line, SC), other osmolytes (orange line), and total osmotic concentration (dashed line). Each point represents the mean ± SE of four irrigated vines at 15:00 in 2021 season. In parenthesis the relative contribution of SC to the osmotic concentration and its statistical significance is written. Different letters represent statistically significant differences between dates (*p* ≦ 0.05, Tukey HSD).

The seasonal increase of *π* was not driven by SC seasonal dynamics (Figure 3). In contrast to the seasonal decrease in SC concentration, the maximum osmotic potential of irrigated vines increased from 405 mmol kg‐1 in April to 535 mmol kg^−1^ by August (Figure 3). At the beginning of the season, between anthesis and veraison, SC comprised ~40% of the total osmotic concentration. In June, between veraison and harvest, the contribution of SC to *π* decreased to 27.4%. After harvest, in August, SC made up only 22.4% of *π*. The concentration of osmolytes other than SC, calculated as the difference between *π*SC and *π*, increased rapidly from 274.5 mmol kg^−1^ (May 31) to 393.7 mmol kg^−1^ just before harvest (June 17). After harvest (June 24), non‐SC osmolytes decreased to 340.5 mmol kg^−1^ (July 06).

### 3.4 Carbohydrates in Relation to Photosynthesis

**FIGURE 4:**
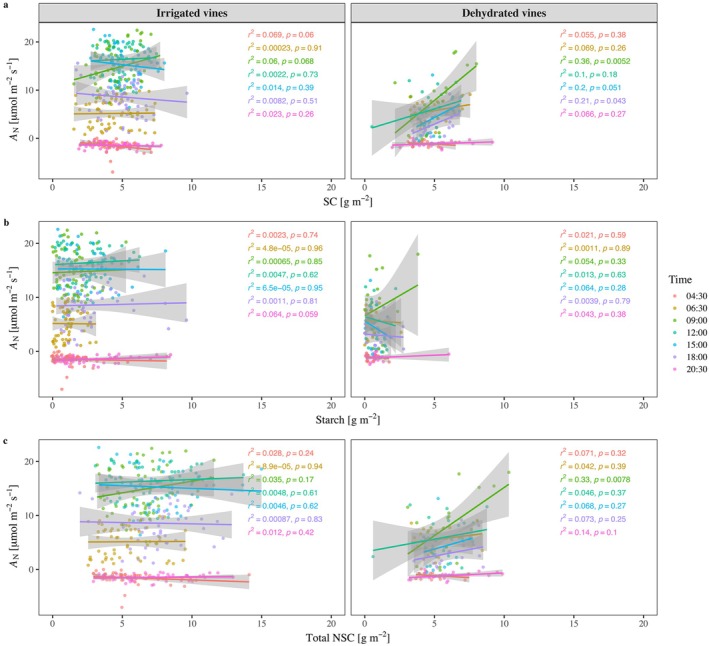
The relationship of soluble carbohydrates (SC, a), starch (b), and total non‐structural carbohydrates (total NSC, c) and carbon assimilation (A_N_) in irrigated and dehydrated vines at different hours.

We used the large dataset we collected to evaluate if photosynthesis and leaf NSC were negatively correlated (i.e., supporting the hypothesis that foliar NSC regulates photosynthesis). The overall regression throughout the two seasons across both irrigation treatments and the various times of day was positive and statistically significant (*p* < 0.001; Figure S5) but very weak (*r* ^2^ = 0.054). The correlation between sugars and *A*_N_ depended on the irrigation treatment and the time of measurement (Figure 4). For irrigated vines, SC~*A*_N_ poorly correlated at all hours (*r* ^2^ < 0.01). However, there was a significant correlation of SC~*A*_N_ at specific times of day in the case of dehydrated vines. For example, the correlation was highest at 09:00 (*r* ^2^ = 0.36, *p* < 0.01) and 18:00 (*r* ^2^ = 0.21, *p* < 0.05). Starch never correlated with *A*_N_ (*r* ^2^ < 0.01), independent of treatment or time (Figure 4a,b). In dehydrated vines, SC fit to *A*_N_ between 09:00 and 18:00 was relatively strong (r^2^ range of 0.1–0.36), but starch ~*A*_N_ *r* ^2^ values were below 0.06 at all hours. Total NSC correlation to *A*_N_ was significant only in dehydrated vines at 09:00 (*r* ^2^ = 0.33, *p* < 0.05; Figure 4c).

## 4 Discussion

Our measurements provide a unique view into the daily and seasonal patterns of NSC dynamics and their high variability. Our results reinforce the importance of NSC to the daily accumulation of osmolytes and the maintenance of positive turgor when *Ψ*_leaf_ is at its minimum. However, our data do not support the hypotheses that SC drives osmotic adjustment under drought or illustrate feedback inhibition by NSC on rates of photosynthesis and leaf gas exchange.

### 4.1 NSC Do Not Take Part in Grapevine Osmotic Adjustment

In agreement with past studies of grapevines, we observed an increase in foliar osmotic concentration in response to water deficit (Patakas et al. 2002; Hochberg et al. 2017; Perry et al. 2025) and as the season progressed (Alsina et al. 2007; Sorek et al. 2021; Herrera et al. 2022; Farolfi et al. 2025). However, SC did not play a major role in these adjustments. In response to water deficit, there was hardly any accumulation of SC in leaves, contrasting previous observations in grapes (Patakas et al. 2002; Hochberg et al. 2013). One explanation for the discrepancy from past studies is that in the current experiment the vines were dried to relatively mild drought stress (*Ψ*_leaf_ > −1.4 MPa) that possibly ignited less osmotic adjustment. At the same time, a recent grapevine experiment that dried vines to significant turgor loss and leaf shedding found that the harsher the drought, the lower the SC concentration in the leaves (Perry et al. 2025). A lack of contribution of SC to osmotic adjustment was also evident in several other species (Lannucci et al. 2002; Ottow et al. 2005). The osmotic adjustments on a seasonal scale were far larger than the osmotic adjustment in response to stress, as was previously noted (Sorek et al. 2021; Carlos Herrera and Hochberg 2024). It is important to note that we measured the solute concentration in non‐rehydrated leaves and thus it could partly be affected by seasonal changes in water content. However, as the seasonal osmotic adjustment was evident in the irrigated plants that constantly maintained high water potential, the effect of the water content was probably marginal. On a seasonal scale, we observed a reduction in leaf SC, despite the increase in *π*. The seasonal decline in SC is probably a function of leaf maturation, as mature grape leaves typically contain lower sugar concentrations than younger leaves (Patakas and Noitsakis 2001). Overall, our data provide evidence that the hypothesis of SC driving osmotic adjustment (Munns and Weir 1981) should not be generalized among taxa.

Moreover, our data challenge previous reports (Rodrigues et al. 1993; Patakas et al. 2002) that SC is a major contributor to grape leaf *π*. We found that SC comprised only 15%–30% of the leaf osmotic content, a proportion significantly lower than the 45%–90% reported by Rodrigues et al. (1993) and Patakas et al. (2002) for grapevines. Interestingly, our raw measurements resemble previous reports of grape leaves (Roper et al. 1989; Williams et al. 2000; Quereix et al. 2001). Thus, we do not think that the discrepancy from Rodrigues et al. (1993) and Patakas et al. (2002) stems from the experimental procedure but from the transformation between dry weight and solute concentrations. Rodrigues et al. (1993) and Patakas et al. (2002) used a very high apoplastic fraction (A_f_ of 60%). Since A_f_ is considered to contain almost no solutes, their estimates of leaf osmotic content assumed that all the SC is dissolved in only 40% of leaf water content. This high A_f_ was evaluated using a pressure‐volume curve, which frequently overestimates the apoplastic fraction by several folds (Arndt et al. 2015). The 10% A_f_ that we used is the average of over a dozen species cross‐validated by two different essays (Tyree 1976; Arndt et al. 2015). A 10% A_f_ also resonates with SEM cross‐sections of grape leaves (Zhang et al. 2022). The resulting proportion of SC as 15%–30% of leaf *π*, which is in line with SC contribution to *π* in many species (e.g., Premachandra et al. 1992; Warren et al. 2012; Gersony et al. 2020), is therefore probably closer to the actual value in grapevines.

### 4.2 Hourly Leaf NSC Dynamics

The daily accumulation of SC did play an important role in maintaining turgor at midday, as formerly demonstrated for corn (Acevedo et al. 1979) and red oaks (Gersony et al. 2020). While the absolute values of this accumulation are not large, they can be crucial for plants to avoid the critical physiological wilting threshold (Bartlett et al. 2014). Since daily sugar accumulation is typical for photosynthesizing leaves (Al‐Sheikh Ahmed et al. 2020), we believe that the phenomenon may be common to many species. Our results demonstrated that leaves under water deficit accumulated similar SC concentrations as watered plants, probably because the dehydrated plants discontinued growth and lowered carbon transport to sinks (Muller et al. 2011). Accordingly, the role of SC in daily osmotic adjustment is also important in stressed plants, where its contribution to turgor is even more critical.

NSC accumulation also affected the diurnal variation in leaf dry mass, although our comparison (Figure 2a) highlighted the importance of other factors. Previous observations for tomatoes (Bertin et al. 1999) and Arabidopsis (Poorter et al. 2009) report that sugars accounted for only 40%–70% of daily LMA changes. The dehydrated vines further highlighted the mismatch between the daily NSC accumulation and LMA buildup. The dehydrated vines showed only 0.5 g m^−2^ lower LMA accumulation along the day than watered vines, yet accumulated 1.5 g m^−2^ less NSC. One explanation is that the leaves, especially those of the dehydrated plants, accumulated other solutes, such as organic acids (Hochberg et al. 2013) or potassium (Degu et al. 2019). Alternatively, leaf shrinkage may have increased LMA. Scoffoni et al. (2014) found that across 15 species, leaf area shrinkage from saturation to turgor loss ranged from 0.5% to 14% (with a 4% average). Based on our LMA measurements, a 1% leaf shrinkage would lead to an LMA increase of 0.4 g m^−2^ and explain much of the daily LMA changes we reported. Greater leaf shrinkage in dehydrated vines could explain their daily increase in LMA despite the low NSC accumulation. The fact that LMA of dehydrated vines started to decrease at 15:00 (Figure 2a) when *Ψ*_leaf_ increased (Figure 1c) also points to the critical role of leaf shrinkage in hourly LMA changes.

An error in our analysis could arise from our non‐specific SC analysis. We assumed that the proportion between sugars was 38% monosaccharides to 62% disaccharides (Rodrigues et al. 1993; Patakas et al. 2002), and we thus used a corresponding molar weight (M_W_) of 280.4 g mol^−1^. Possibly, sugar proportions differed for the current study, perhaps even changing between hourly measurements or along the season, and it is important to evaluate such a potential bias. If monosaccharides presented 90% of the total SC (M_W_ = 196.4 g mol^−1^), then SC's contribution to the overall osmotic potential would increase to 32%–58% (from 22% to 40% in the current analysis). Likewise, SC contribution to the daily osmotic accumulation would increase to 72% (from 50.9%) in the dehydrated and 46.1% (from 32.3%) in the watered vines. If monosaccharides presented 10% of the total SC (M_W_ = 326.1 g mol^−1^), SC contribution to the overall osmotic potential would diminish to 19%–34%, and its contribution to the daily osmotic accumulation would decrease to 43.8% in the dehydrated vines and 27.8% in the watered vines. This bias control demonstrates that the conclusion of the current analysis will not significantly change even if we wrongly estimated the average M_W_ of the SC. It is important to mention that our main conclusion regarding the lack of SC importance to osmotic adjustments is based on the fact that the SC did not accumulate following dehydration and would not have changed regardless of the sugars' exact M_W_.

### 4.3 The Regulation of Carbon Assimilation Is Not Signaled by NSC

We collected a large dataset of 840 coupled *A*_N_ and NSC measurements, which enabled us to test the hypothesized causality between NSC content and the regulation of photosynthesis. The correlations between *A*_N_ and NSC did not show any inhibitory effect of NSC on photosynthesis, occasionally (at 9:00–15:00) even demonstrating a positive correlation (Figure 4). The fact that photosynthesis is the source for NSC could explain the positive correlation. Overall, our data do not support the formerly acclaimed hypothesis regarding the end‐product inhibition of photosynthesis (Boussingault 1868).

However, there are several limitations to our study that could limit our conclusions. For starters, our vines were relatively young (3–4 years old) and had low yields (4–7.5 kg vine^−1^ compared with > 20 kg vine^−1^ in a well‐developed vine from this cultivar). The low fruit load and its subsequential low carbon sink could hinder the interaction between leaf NSC and gas exchange. In a similar manner, Sperling et al. (2024) found a strong effect of potassium on g_s_ under high fruit load, but not under low fruit load. Further, it is impossible to negate relationships based on weak correlations. A small signal‐to‐noise ratio might result in a bias, leading to the rejection of existing links. To diminish the potential noise, we analyzed the relationships by different treatments and time of day (Figure 4). We have also considered the possibility of a lag between NSC accumulation and gas exchange downregulation. Still, correlations remained insignificant (Figure S6). We also verified that our signal (i.e., NSC and *A*_N_ changes) was as substantial as those in studies that found a negative correlation between these parameters (Williams et al. 2000; Tombesi et al. 2019; Dayer et al. 2021). Accordingly, we think the current findings strongly undermine the possibility that bulk leaf NSC signal photosynthetic inhibition. A recent publication that followed the NSC and gas exchange dynamics of 114 species reached a similar conclusion (Asao et al. 2025).

Past studies that advocated for end‐product inhibition of photosynthesis relied on source‐sink manipulation (e.g., girdling and fruit removal), assuming causality between the NSC content and the supposedly consequential change in *A*_N_ (e.g., Goldschmidt and Huber 1992). Such studies with similar findings were also conducted on grapevines (Kriedemann and Lenz 1972; Roper et al. 1989; Williams et al. 2000), highlighting that genotypic differences do not drive the discrepancy with the current findings. We should consider the possibility that the experimental manipulations triggered the downregulation of *A*_N_ by signals other than NSC. For example, hormonal signals are sharply altered following girdling (Castro‐Valdecantos et al. 2021). Mitchell et al. (2017) found that both NSC and abscisic acid increased 22 days after girdling, along with a sharp decline in gas exchange. However, 35 days after girdling, NSC were lower than the pre‐girdling values, while gas exchange rates kept declining following further accumulation of ABA. A relatively slow NSC increase compared to the rapid decline in gas exchange after girdling (Nebauer et al. 2011) further reinforces our notion that sugars are not the main signals for photosynthetic inhibition.

Importantly, our findings do not negate the link between carbon source‐sink relations and photosynthesis but demonstrate that the primary signal is not the leaf bulk NSC content. Quereix et al. (2001) suggested that the phloem or the sugar concentrations in sink tissues are better candidates as markers for the source‐sink relation, though they did not indicate how these signals are transmitted to the chloroplasts. Herold (1980) and Plaut et al. (1987) argued that the chloroplast could sense the source‐sink relation through levels of inorganic phosphorus or phosphorylated compounds, as these are related to sugar metabolism and can easily cross the chloroplast membrane. To date, there is not sufficient data to determine which molecules signal the inhibition of photosynthesis.

To conclude, our findings challenge both of the study's hypotheses. We failed to show that leaf SC is critical for osmotic adjustment in grapevines exposed to water deficits. This was even clearer on a seasonal level, where the osmotic importance of SC decreased throughout the growing season while osmotic concentration increased. Similarly, the lack of correlations between NSC and *A*_N_ challenges the hypothesis of a simple end‐product inhibition of photosynthesis. This conclusion requires further investigation, as the bulk leaf NSC content is not necessarily a good representation of the end product. At the same time, hourly SC accumulation in dehydrated grapevines appears critical for turgor maintenance at midday when *Ψ*_leaf_ is lowest.

## Author Contributions

Aviad Perry, Or Sperling, N. Michele Holbrook, Alon Ben‐Gal, and Uri Hochberg designed the experiment and the methodological setup. Aviad Perry carried out the experiment, analysis of the results, and preparation of the figures with the supervision of Shimon Rachmilevitch, Or Sperling, and Uri Hochberg. The first draft of the manuscript was written by A.P. and U.H. with major contributions from all authors.

## Funding

This research was supported by the Israel Ministry of Agriculture, Chief Scientist Research Grant no. 20‐03‐0044 (http://www.moag.gov.il/agri/yhidotmisrad/madanrashi/); and by Grant no. 2795‐18 from BARD, the United States‐Israel Binational Agricultural and Development Fund (http://www.bard‐isus.com/). Aviad Perry was supported by the “STEP‐GTP – science training encouraging peace‐ graduate training program” fellowship.

## Conflicts of Interest

The authors declare no conflicts of interest.

## Supporting information


**Data S1:** Supplementary Information.

## Data Availability

The data that supports the findings of this study can be obtained upon reasonable request from the corresponding author Uri Hochberg.
