# Supplementary material for: Do Daily and Seasonal Changes in Non‐Structural Carbohydrates in Grapevine Leaves Contribute to Osmotic Adjustment and Regulation of Photosynthesis?
Source: Physiol Plant. 2025 Dec 29;178(1):e70683. doi: 10.1111/ppl.70683 (PMC12746217; doi:10.1111/ppl.70683)
Supplement: Supplementary file 1 — Data S1: Supplementary Information. [file PPL-178-e70683-s001.docx]

**Do daily and seasonal changes in non-structural carbohydrates in grapevine leaves contribute to osmotic adjustment and regulation of photosynthesis?**

**Aviad Perry^1^**[
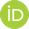
](https://orcid.org/0000-0002-4975-1724)**, Or Sperling^2^**[
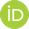
](https://orcid.org/0000-0002-4007-4341)**, Alon Ben-Gal^3^**[
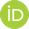
](https://orcid.org/0000-0003-4105-7807)**, N. Michele Holbrook^4^**[
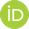
](https://orcid.org/0000-0003-3325-5395)**, Shimon Rachmilevitch^5^**[
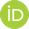
](https://orcid.org/0000-0003-3600-5949)**, and Uri Hochberg^6^**[
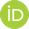
](https://orcid.org/0000-0002-7649-7004)

^1^Kreitman School for Graduate Studies, Ben-Gurion University of the Negev, Israel

^2^Plant Sciences, Agricultural Research Organization-Volcani Institute, Gilat Research Center, Israel

^3^Soil, Water and Environmental Sciences, Agricultural Research Organization-Volcani Institute, Gilat Research Center, Israel

^4^Department of Organismic and Evolutionary Biology, Harvard University, Cambridge, Massachusetts, USA

^5^French Associates Institute for Agriculture and Biotechnology of Drylands, The Jacob Blaustein Institutes for Desert Research, Ben Gurion University of the Negev, Sede Boqer, Israel

^6^Institute of Soil, Water, and Environmental Sciences, Agricultural Research Organization-Volcani Institute, Neve Ya'ar research center, Israel

**Correspondence:**

*Corresponding authors,

E-mail: hochberg@volcani.agri.gov.il

# **Supplementary data**

**Table S1.** Dates of diurnal measurements in seasons of 2020 and 2021. At dates of dehydration trials, the dehydration process duration prior to the measurement is mentioned in days inside the parenthesis.

| **Season** | **Date** | **Dehydration** |
| --- | --- | --- |
| 2020 | 13-May |  |
|  | 27-May |  |
|  | 03-Jun | V (6) |
|  | 18-Jun |  |
|  | 01-Jul |  |
|  | 09-Jul | V (6) |
|  | 22-Jul |  |
|  | 20-Aug | V (9) |
| 2021 | 28-Apr |  |
|  | 12-May |  |
|  | 31-May | V (6) |
|  | 17-Jun |  |
|  | 06-Jul | V (7) |
|  | 26-Jul |  |
|  | 10-Aug | V (7) |

**Table S2.** Type III analysis of variance (ANOVA) for linear mixed-effects models testing the effects of measurement time, treatment, and season (2020 vs. 2021) on leaf physiological traits. Response variables included assimilation rate (A_N_), stomatal conductance (g_s_), leaf water potential (Ψ_leaf_), soluble carbohydrate (SC), starch and osmotic concentration (π). All these traits were modeled with *time*, *treatment*, and *season* as fixed factors, and *date* as a random intercept to account for repeated measurements within the same day. For π, which was only measured in 2021, the model included *time* and *treatment* as fixed factors, with *date* as a random intercept. Reported are numerator and denominator degrees of freedom (NumDF, DenDF), F-statistics, and P-values, based on Type III sums of squares. Significance levels: *** P ≤ 0.001; ** P ≤ 0.01; * P ≤ 0.05.

| **Variable** | **term** | **sumsq** | **meansq** | **NumDF** | **DenDF** | **statistic** | **P-value** |
| --- | --- | --- | --- | --- | --- | --- | --- |
| A_N_ | time | 9190.24 | 1531.71 | 6 | 484.17 | 263.41 | <0.001 *** |
|  | treatment | 1356.88 | 1356.88 | 1 | 495.96 | 233.34 | <0.001 *** |
|  | season | 26.01 | 26.01 | 1 | 12.99 | 4.47 | 0.054 |
|  | time:treatment | 2314.16 | 385.69 | 6 | 484.09 | 66.33 | <0.001 *** |
|  | time:season | 460.46 | 76.74 | 6 | 484.17 | 13.20 | <0.001 *** |
|  | treatment:season | 151.61 | 151.61 | 1 | 495.96 | 26.07 | <0.001 *** |
|  | time:treatment:season | 130.70 | 21.78 | 6 | 484.09 | 3.75 | 0.001 ** |
| g_s_ | time | 2279159.93 | 379859.99 | 6 | 483.91 | 69.36 | <0.001 *** |
|  | treatment | 969678.84 | 969678.84 | 1 | 494.68 | 177.04 | <0.001 *** |
|  | season | 0.02 | 0.02 | 1 | 12.87 | 0.00 | 0.998 |
|  | time:treatment | 1330697.89 | 221782.98 | 6 | 483.80 | 40.49 | <0.001 *** |
|  | time:season | 41942.21 | 6990.37 | 6 | 483.91 | 1.28 | 0.267 |
|  | treatment:season | 29816.56 | 29816.56 | 1 | 494.68 | 5.44 | 0.020 * |
|  | time:treatment:season | 49343.99 | 8224.00 | 6 | 483.80 | 1.50 | 0.176 |
| Ψ_leaf_ | time | 4210.02 | 701.67 | 6 | 483.91 | 547.76 | <0.001 *** |
|  | treatment | 380.59 | 380.59 | 1 | 495.18 | 297.11 | <0.001 *** |
|  | season | 9.05 | 9.05 | 1 | 12.84 | 7.07 | 0.020 * |
|  | time:treatment | 70.98 | 11.83 | 6 | 483.81 | 9.24 | <0.001 *** |
|  | time:season | 86.06 | 14.34 | 6 | 483.91 | 11.20 | <0.001 *** |
|  | treatment:season | 52.86 | 52.86 | 1 | 495.18 | 41.27 | <0.001 *** |
|  | time:treatment:season | 64.94 | 10.82 | 6 | 483.81 | 8.45 | <0.001 *** |
| SC | time | 30.22 | 5.04 | 6 | 484.06 | 5.16 | <0.001 *** |
|  | treatment | 0.02 | 0.02 | 1 | 494.34 | 0.02 | 0.881 |
|  | season | 1.22 | 1.22 | 1 | 12.58 | 1.25 | 0.285 |
|  | time:treatment | 7.10 | 1.18 | 6 | 484.01 | 1.21 | 0.299 |
|  | time:season | 5.34 | 0.89 | 6 | 484.06 | 0.91 | 0.485 |
|  | treatment:season | 1.07 | 1.07 | 1 | 494.34 | 1.10 | 0.295 |
|  | time:treatment:season | 2.05 | 0.34 | 6 | 484.01 | 0.35 | 0.91 |
| Starch | time | 63.84 | 10.64 | 6 | 484.03 | 9.58 | <0.001 *** |
|  | treatment | 137.40 | 137.40 | 1 | 495.89 | 123.75 | <0.001 *** |
|  | season | 1.40 | 1.40 | 1 | 12.74 | 1.26 | 0.282 |
|  | time:treatment | 34.55 | 5.76 | 6 | 483.96 | 5.19 | <0.001 *** |
|  | time:season | 16.57 | 2.76 | 6 | 484.03 | 2.49 | 0.022 * |
|  | treatment:season | 38.31 | 38.31 | 1 | 495.89 | 34.50 | <0.001 *** |
|  | time:treatment:season | 5.42 | 0.90 | 6 | 483.96 | 0.81 | 0.559 |
| π | time | 122545.90 | 20424.32 | 6 | 201.94 | 3.62 | 0.002 ** |
|  | treatment | 12408.48 | 12408.48 | 1 | 206.60 | 2.20 | 0.14 |
|  | time: treatment | 15118.76 | 2519.79 | 6 | 201.94 | 0.45 | 0.847 |


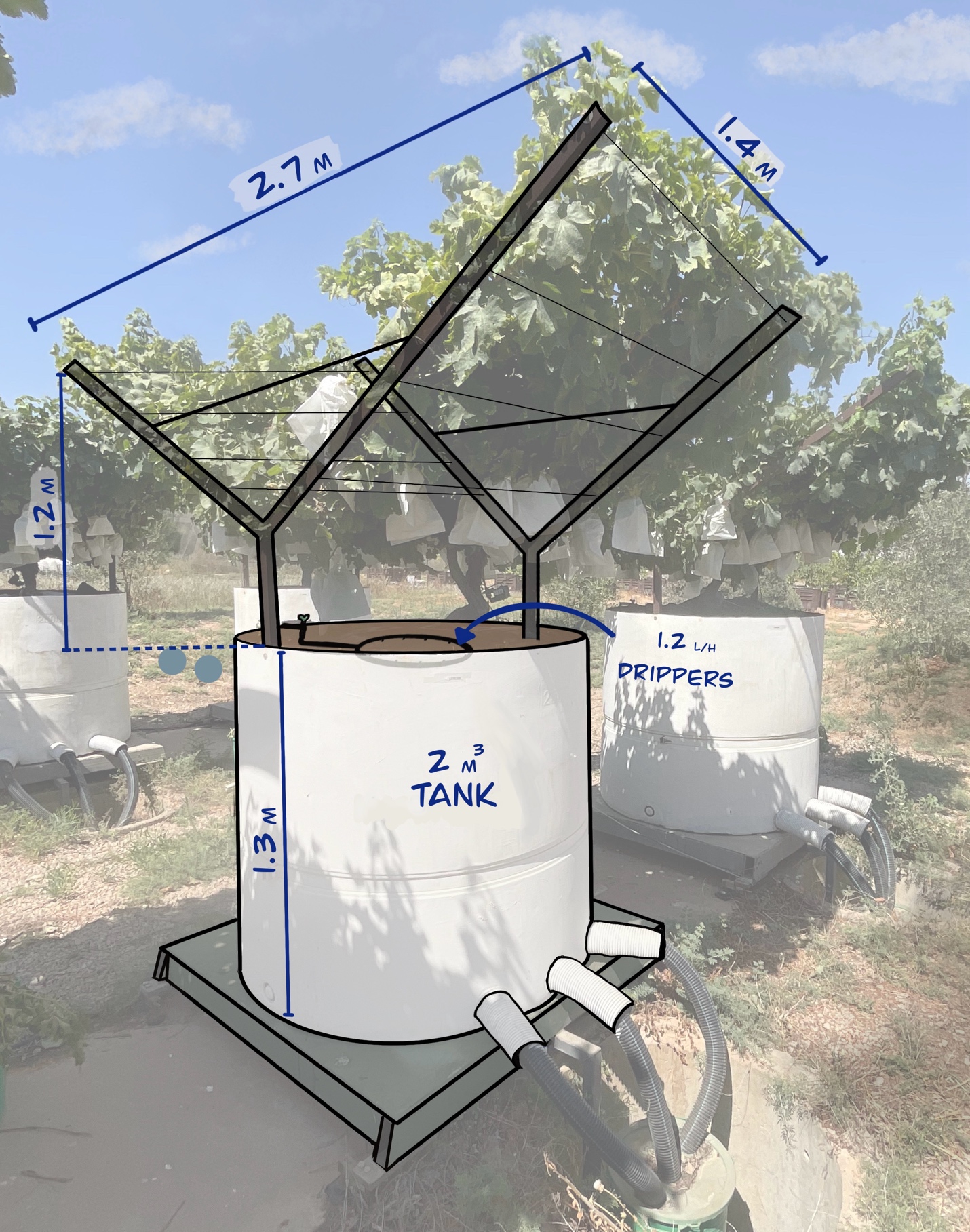


**Figure S1.** A schematic description of the tank, the vine and the trellis system.

**Figure S2.** Daily potential evapotranspiration (ET_0_) and dates of physiological and metabolic measurements (arrows) in 2020 (a), and 2021 (b). Yellow area represents days of drought trials. Red arrows represent days where both irrigated dehydrated vines were measured.

**Figure S3.** The maximal and minimal temperatures during the growth seasons in 2020 (a) and 2021 (a).

**Figure S4.** The photosynthetic active radiation (PAR) and relative humidity (RH) during the growth seasons in 2020 and 2021.

**Figure S5.** The diurnal relationship of soluble carbohydrates (SC, a), starch (b), and total non-structural carbohydrates (total NSC, c) and carbon assimilation (*A*_N_) across both irrigation treatments and all hours.

**Figure S6.** The relationship of soluble carbohydrates (SC, a), starch (b), and total non-structural carbohydrates (total NSC, c) and stomatal conductance (g_s_) in irrigated and dehydrated vines at different hours.
